# Citrullinemia type I in Chinese children: Identification of two novel argininosuccinate synthetase gene mutations

**DOI:** 10.3389/fped.2022.992156

**Published:** 2022-10-03

**Authors:** Mei Xiong, Mingwu Chen

**Affiliations:** Department of Pediatrics, The First Affiliated Hospital of USTC, Division of Life Sciences and Medicine, University of Science and Technology of China, Hefei, China

**Keywords:** argininosuccinate synthetase gene, novel mutations, citrullinemia type I, tandem mass spectrometry, whole-exome sequencing

## Abstract

**Background:**

In this study, we evaluated the clinical characteristics, prognosis, and gene mutations of five children with citrullinemia type I (CTLN1) diagnosed in our department and identified two novel ASS1 gene mutations.

**Methods:**

We examined the clinical characteristics, prognosis, and gene mutations of the five children through data collection, tandem mass spectrometry, and whole-exon sequencing. MutationTaster, regSNP-intron, and SWISS-MODEL were used for bioinformatic analysis to evaluate the two novel gene mutations. We analyzed differences in blood ammonia and citrulline levels based on clinical phenotypes. Finally, we reviewed the medical literature describing Chinese children with CTLN1.

**Results:**

ASS1 C773 + 6T > G and c.848 delA as well as c.952_953 del insTT and c.133G > A have not been previously reported in the Human Gene Mutation Database. Using MutationTaster and regSNP-intron, we predicted that these mutations affected protein function. The 3D structure obtained using SWISS-MODEL supported this prediction. Through comparative analysis showed that the ammonia level of the neonatal type was markedly higher than that of other types, whereas citrulline levels did not differ between groups.

**Conclusion:**

We identified two novel mutations that cause disease. The blood ammonia level of neonatal form citrullinemia was markedly higher than that of other types. The genotype-phenotype association in Chinese patients remains unclear and should be further evaluated in genetic studies of larger sample sizes.

## Introduction

Citrullinemia type I (CTLN1) is a rare congenital metabolic disease (1 in 250,000 live births) caused by mutations in the argininosuccinate synthetase (*ASS1*) gene (1, 2). The resulting enzyme deficiency leads to high concentrations of ammonia and citrulline (3) in the blood and plasma. Based on the different functions of *ASS1*, CTLN1 can be divided into four clinical phenotypes: acute neonatal, mild later-onset, asymptomatic, and an additional form in which women express symptoms during pregnancy (4). The virulence *ASS1* of CTLN1 is in 9q43.1, and numerous mutations have been identified in *ASS1* (5). For example, the mutation p.Gly390Arg (5) is commonly detected worldwide. In Korea (6), the most widely distributed variation is the p.Gly324Ser mutation, which has been reported in many countries such as America and Germany but has not been reported in Japan. The c.421-2A > G mutation (7) is very common in Japan. In German patients, the p.Arg363Trp mutation is the most common mutation.

In this study, we evaluated the clinical features, laboratory data, outcomes, and genetic characteristics of five children with CTLN1, in whom two novel mutations were identified. Moreover, using the keyword CTLN1, we performed literature searches and summarized the previously reported characteristics of Chinese children with CTLN1 to improve the understanding of this disease in the Chinese population.

## Materials and methods

### Subjects

From August 2020 to February 2022, five inpatients with CTLN1 in the First Affiliated Hospital of Science and Technology of China participated in this study. Informed consent was obtained from all guardians of the participants, and the patients were diagnosed based on their clinical characteristics, laboratory data, tandem mass spectrometry, and gene mutation analysis. Blood samples were obtained for genetic analysis, and blood and urine were evaluated in tandem mass spectrometry analysis. This study was approved by the medical research ethics committee of our hospital. We also reviewed previously published literature on children from China with CTLN1.

### Tandem mass spectrometry analysis of blood and urine

Non-anticoagulant whole blood or urine was collected and spotted onto dry blood filter paper by the Chief Resident at the First Affiliated Hospital of Science and Technology of China. Three blood spot specimens were collected from each patient, cooled, dried at 15–22°C, and transferred to the screening laboratory at 3–9°C. The derivative reagent (60 μL of 3 mmol/L *n*-butyl acetate) was added to U-bottom plates. After incubation for 35 min at 40°C, 70 μL of the solution in the plates was transferred to new plates. The liquid was left standing for 2.5 h at 20–25°C, and then 30 μL of liquid was collected for metabolite analysis *via* tandem mass spectrometry on an instrument from AB SCIEX (Framingham, MA, USA). The *n*-butyl acetate, amino acid internal standard, acylcarnitine internal standard, carbinol, and other reagents were purchased from Guangzhou Jin Xinrui Biotechnology Co., Ltd. (Guangzhou, China).

### Direct sequencing analysis of argininosuccinate synthetase

#### Target region capture and sequencing

Peripheral blood (2 mL) was drawn from the subjects and controls. Genomic DNA was extracted using a MagPure Buffy Coat DNA Midi KF Kit (Beijing Genomics Institute, Beijing, China) according to the manufacturer’s instructions. Genomic DNA was cleaved into 100–500 bp fragments using a Segmentase enzyme kit (BGI, Cambridge, MA, USA), and then 280–320 bp fragments were collected using magnetic beads. In the collection, the “A” base was added at the 3’ overhangs after end repair to ensure that the fragments could pair with the “T” base on the special adapter. A single individual DNA library was prepared after ligation-mediated polymerase chain reaction and purification. The library was enriched for 16–24 h (47°C) by array hybridization (Roche NimbleGen, Basel, Switzerland), followed by elution and post-capture amplification. The products were evaluated using a 2100 Bioanalyzer (Agilent Technologies, Santa Clara, CA, USA) to estimate the magnitude of enrichment. Qualified products were pooled and quantified according to the different library quantities. Single-strand library products were prepared for circularization and to prepare DNA nanoballs. The products were sequenced to obtain the 100 bp paired-end reads on an MGISEQ-2000 (MGI Tech Co., Ltd., Mogi das Cruzes, Brazil) This strategy first generates a 100 bp first-end read and then generates a 100 bp second-end read. The detailed 100 bp pair-end sequencing steps performed on the DNBSEQ MPS platform were as follows. A DNA nanoball, as a concatemer containing copies of the adaptor sequence and inserted genomic DNA, was hybridized with a primer for first-end sequencing. After generating the first-end read (100 bp), controlled and continued extension was performed by strand-displacing DNA polymerase to generate complementary strands. When the 3’ ends of the newly synthesized strands reached the 5’ ends of the downstream strands, the 5’ ends were displaced by DNA polymerase to generate single-stranded DNA overhangs, creating a “branched DNA nanoball.” A second-end sequencing primer was hybridized to the adaptor copies in the newly created branches to generate a second-end read (100 bp).

#### Sanger sequencing (whole-exome sequencing)

The Sanger sequencing workflow involved amplifying the sequence by PCR and performing sequencing and capillary electrophoresis. During PCR and cycle sequencing, the DNA was denatured into single strands. Annealing was performed to hybridize the oligonucleotide primer close to the sequence of interest. In the extension step, the DNA polymerase extended the primer from its 3’ hydroxyl group to synthesize a new strand. An adenine base (A) was paired with each thymine (T) on the template and a cytosine (C) with each guanine (G), and vice versa. Occasionally, one of the four chain-terminating ddNTPs was inserted by chance, which interrupted elongation of the DNA strand. The elongation reaction was repeated for 30–40 cycles.

Following obtaining clean sequencing reads, the newly synthesized DNA fragments were separated by electrophoresis in a single long glass capillary filled with a gel polymer. Using an optimized combination of a very thin capillary, appropriate choice of gel or polymer, and electric field parameters, capillary electrophoresis separated DNA strands up to ∼1,000 bp in length at single-nucleotide resolution.

When the fragments migrated through the capillary, a laser excited the fluorescent label on the ddNTP incorporated at the end of each terminated chain. Because each of the four ddNTPs was labeled with a different color, the signal emitted by each excited nucleotide corresponded to a specific base. Software generated a chromatograph showing the fluorescent peak of each labeled fragment.

### Data analysis

After sequencing, the raw data were saved in FASTQ format. Both MGI sequencing adapters and low-quality reads (<80 bp) were filtered using cutadaptor software.^1^ The clean reads were mapped to the UCSC hg19 human reference genome using the BWA parameter in Sentieon software.^2^ Duplicate reads were removed using the parameter driver in Sentieon, and base correction was performed such that the quality value for the reads of the final output BAM file were close to the probability of mismatch with the reference genome. The mapped reads were used for variation analysis. Single-nucleotide polymorphism and insertion-deletion variants were detected using the parameter driver in Sentieon. The data were transformed into VCF format. Variants were further annotated using ANNOVAR software^3^ and searched in multiple databases, including 1000 Genome, ESP6500, dbSNP, EXAC, Inhouse (MyGenostics), and Human Gene Mutation Database. Further comparisons were performed in SIFT, PolyPhen-2, MutationTaster, and GERP3.

### Sanger verification method

All mutations and potential pathogenic variants were validated using conventional Sanger sequencing methods. Segregation analysis was performed when DNA from the family members was available.

### Functional analyses of novel mutations

To validate the effect of the mutations on protein function, MutationTaster, RegSNP-intron, and SWISS-MODEL^4^ were used to conduct bioinformatics analysis. The 3D structures of wild-type and mutant proteins were constructed, and we predicted that disruption of the mutation caused the disease.

## Results

### Patient characteristics and gene mutation spectrum

Using the Human Gene Mutation Database, we identified two novel mutations in the five patients based on genetic mutation analysis. The characteristics and mutation spectra of the five patients are described below.

**FIGURE 1:**
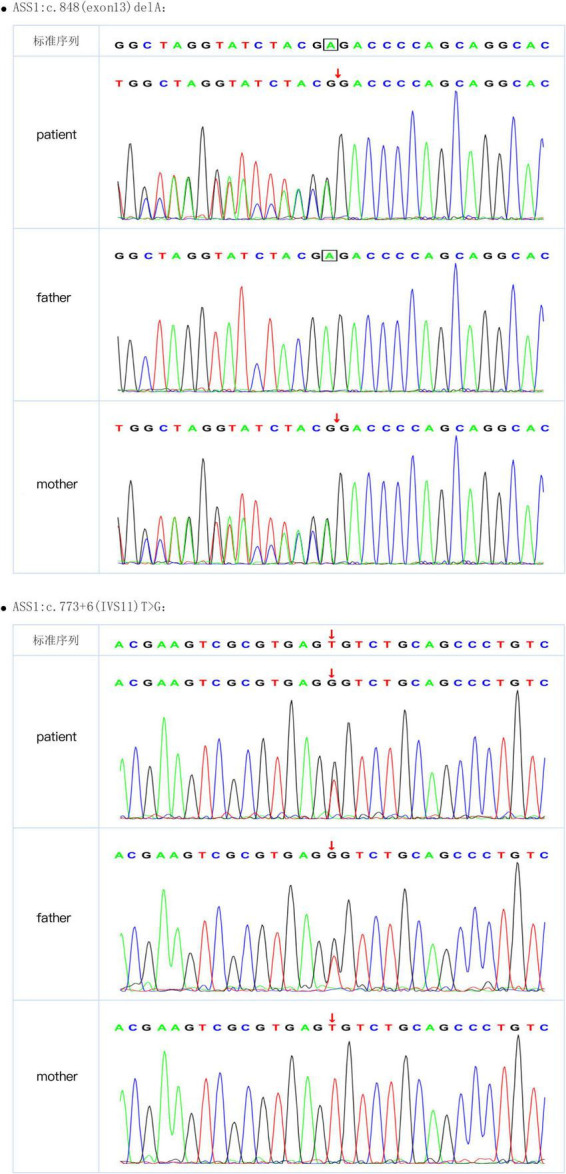
Sanger sequence of *ASS1* (c.848 delA and c.773 + 6T > G) from patient 1 and her parents.

After a 40-week gestation period, patient 1 was vaginally delivered, weighed 3,330 g, and had a body-length of 49 cm and a head circumference of 34 cm. Her parents were healthy; she had amniotic fluid pollution of degree 3 and no clinical symptoms at birth. Nevertheless, on day 6, she experienced feeding difficulties and vomiting. Plasma ammonia levels were markedly increased (424 μmol/L) Because of the elevated plasma ammonia levels, feeding difficulties and vomiting, we analyzed the blood using tandem mass spectrometry, which revealed elevated citrulline levels. The patient was diagnosed with CTLN1 through gene mutation analysis, which revealed the presence of the *ASS1* variants c.848 delA and c.773 + 6T > G (Figure 1). These variations have not been reported previously in the Human Gene Mutation Database. After admission, the patient was administered L-carnitine, and the plasma ammonia level (124 μmol/L) markedly declined. At 7 months, solid food was appended, ammonia levels markedly increased, and the patient began vomiting excessively, likely because of the elevated plasma ammonia level (200 μmol/L). Finally, the patient underwent liver surgery transplantation. She subsequently became healthy.

**FIGURE 2:**
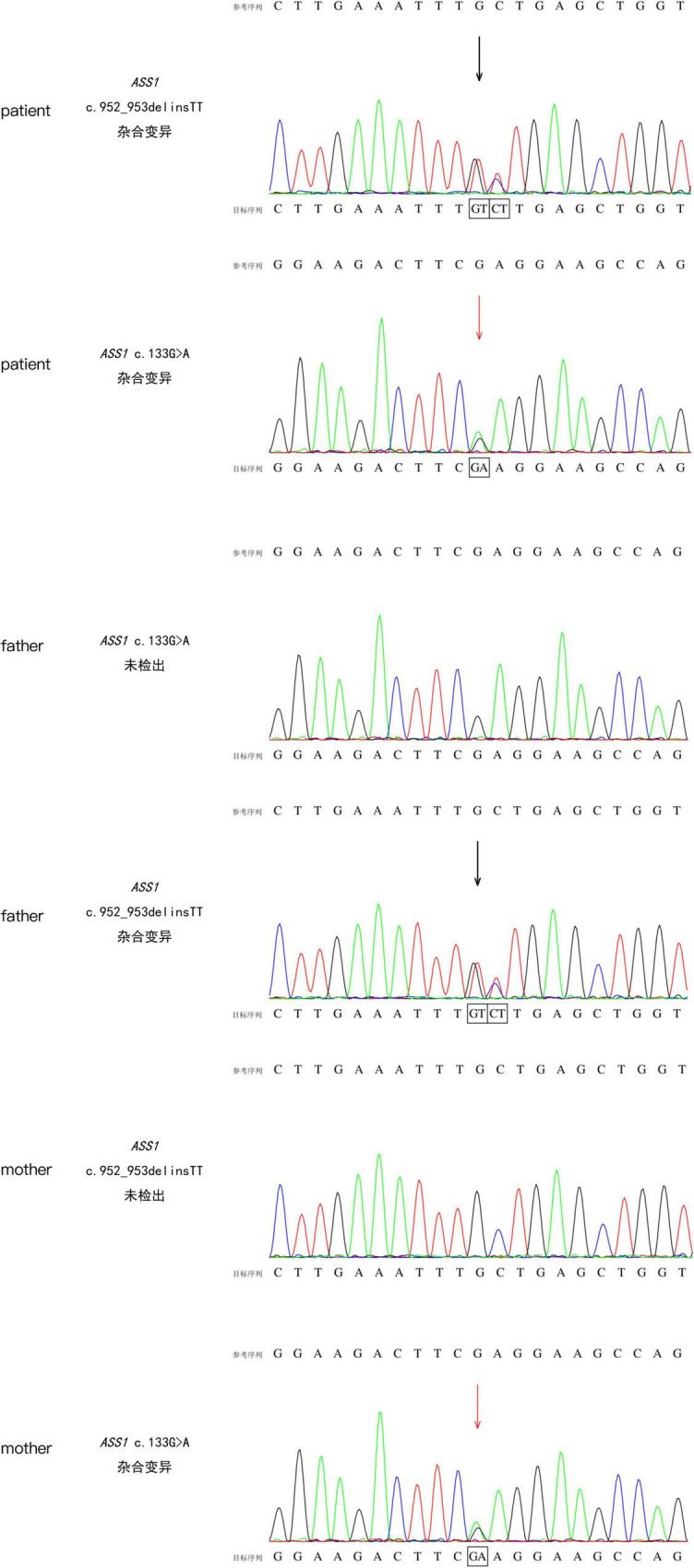
Sanger sequence of *ASS1* (c.952_953 delinsTT and c.133G > A) from patient 2 and her parents.

Patient 2 was born at a gestational age of 33 weeks and 3 days and weighed 2,100 g. Routine new-born blood amino acid screening detected increased citrulline (66 μmol/L, RR, 9.0–33 mmol/L) and glutamic-pyruvic transaminase levels (191 IU/L). The patient was diagnosed with CTLN1 based on tandem mass spectrometry of the blood and the gene mutation spectrum, which revealed the presence of the *ASS1* variant c.952_953 delinsTT and c.133G > A (Figure 2). These variations have not been reported previously in the Human Gene Mutation Database. The patient was not treated because of the absence of symptoms.

Patient 3 was delivered *via* cesarean section at a 39-week gestational age, weighed 2,800 g, and had a length of 49 cm and head circumference of 34 cm. Unfortunately, her sister died 10 years prior but had no clinical symptoms at birth. On day 5, the patient exhibited impaired consciousness and hyperspasmia. Plasma ammonia levels were markedly increased (848 μmol/L) Considering these symptoms, we analyzed the blood and urine using tandem mass spectrometry, which revealed elevated citrulline levels. Gene mutation analysis revealed the presence of the *ASS1* variants c.577 G > A and c.552 C > A; the patient was diagnosed with CTLN1. After admission to the hospital, the patient was administered L-carnitine and mechanical ventilation. The plasma ammonia level significantly increased (2,840 μmol/L). Ultimately, the parents withdrew treatment, and, unfortunately, the patient died.

Patient 4 was delivered at a gestational age of 40 weeks and 2 days *via* cesarean section, weighed 2,800 g, and had a length of 49 cm and head circumference of 34 cm; the patient exhibited no clinical symptoms. On day 7, the patient showed impaired consciousness and hyperspasmia. The plasma ammonia levels were markedly elevated (1,012 μmol/L). Tandem mass spectrometry analysis of the blood and urine, which revealed a significantly elevated citrulline level, confirmed the CTLN1 diagnosis. However, her gene mutation spectrum was not determined because she underwent red cell transfusion. After admission, the patient was administered blood purification and mechanical ventilation. Unfortunately, the parents withdrew treatment, and the patient died.

Patient 5 was delivered at a gestational age of 41 weeks and 1 day and weighed 4,170 g. Routine new-born blood amino acid screening detected elevated ammonia (196 μmol/L, RR, 9.0–33 mmol/L) and citrulline levels (2,525 IU/L). Tandem mass spectrometry of the blood and a gene mutation spectrum, which revealed the *ASS1* variants c.1168 G > A and c.970 G > A, confirmed the CTLN1 diagnosis. For treatment, the patient was placed on a diet of low-protein milk powder. At 2 years of age, the patient exhibited no symptoms.

The characteristics and gene mutation spectra of the five patients are summarized in Tables 1, 2. As shown in Table 1, the clinical manifestations were mostly vomiting or feeding difficulties along with conscious disturbance or hyperspasmia. Disease severity was closely related to the ammonia levels with higher blood ammonia levels associated with more severe symptoms.

**TABLE 1:** Clinical, laboratory data, and outcomes of the five patients.

Patient no.	Sex	Age of onset	Clinical presentation	Conscious disturbance or hyperspasmia	Vomit or feeding difficulties	Highest level of ammonia (μ mol/L)	Highest level of citrulline (μ mol/L)	Outcome
1	F	6 days	Milder later-onset form	No	Yes	424	231	Live by liver transplantation
2	M	Neonatal screening	Without symptoms	No	No	17.2	66.03	Live with no treatment
3	F	5 days	Acute neonatal form	Yes	Yes	2,840	1,731	Died
4	M	7 days	Acute neonatal form	Yes	Yes	1,012	1,689	Died
5	M	Neonatal screening	Without symptom	No	No	196	2,525	Live by low protein milk powder

**TABLE 2:** Gene mutations characteristics of the five patients.

Patient no.	Novel mutation	Clinical presentation	Mutation 1		Mutation 2	
			cDNA	Protein	cDNA	Protein
1	Yes	Milder later-onset form	c.848 del A	p.Glu283 Glyfs*13	c.773 + 6 T > G	–
2	Yes	Without symptoms	c.952_953 del insTT	Glu45Lys	c.133 G¿A	Ala318Phe
3	No	Acute neonatal form	c.577 G > A	Gly193Arg	c.552 C > A	Asn184Lys
4	No	Acute neonatal form	None	None	None	None
5	No	Without symptom	c.1168 G > A	Gly390Arg	c.970G > A	Gly324Ser

### Tandem mass spectrometry analyses of blood and urine

The tandem mass spectrometry characteristics are summarized in Table 3. During the urea cycle, because of a deficiency or hypo-functionality of ASS1, citrulline (substrate) levels increased and arginine (product) levels declined. However, these changes had no effect on fatty acid or carnitine metabolism, as demonstrated by normal blood and plasma acylcarnitine levels. The increased citrulline levels affected pyrimidine-nucleotide synthesis, which increased urinary orotic acid levels.

**TABLE 3:** Tandem mass spectrometry characteristics of the five patients.

Patient no.	Blood/Plasma ammonia (μ mol/L) [0–33]	Blood/Plasma citrulline (μ mol/L) [5–40]	Blood/Plasma arginine μ mol/L [0–50]	Blood/Plasma acylcarnitines (μ mol/L) [10–50]	Urine orotic acid (μ mol/L) [0–2]	Urine lactate (μ mol/L) [0–12]
1	424	231	7	12	12	1
2	17	66	16	28	2	3
3	2,840	1,731	5	17	131	2
4	1,012	1,689	6	15	27	3
5	196	2,525	7	35	0	1

### Functional analyses of novel mutations

Analysis by MutationTaster and regSNP-intron suggested that the variants likely affected protein function (Table 4). Based on American College of Medical Genetics guidelines, the novel gene mutations were classified as variants of clinical significance.

**TABLE 4:** Effect of novel gene mutation on protein function according to *in silico* analysis.

cDNA	Protein	Software (score)		Predicted signal
		MutationTaster	RegSNP-intron	
c.848delA	p.E283Gfs*1 3(p.Glu283 Glyfs*13)	**1**	–	Disease_causing
c.773 + 6T > G	–		0.74	–
c.133G > A	Glu45Lys	0.99	–	Disease_causing
c.952_953delinsTT	Ala318Phe	0.99	–	Disease_causing

MutationTaster, www.mutationtaster.org. Scores between 0 and 1; a score closer to 1 indicates that the mutation is disease-causing. regSNP-intron, http://regsnps-intron.ccbb.iupui.edu/. Scores between 0 and 1; a score closer to 1 indicates that the mutation is disease-causing.

**FIGURE 3:**
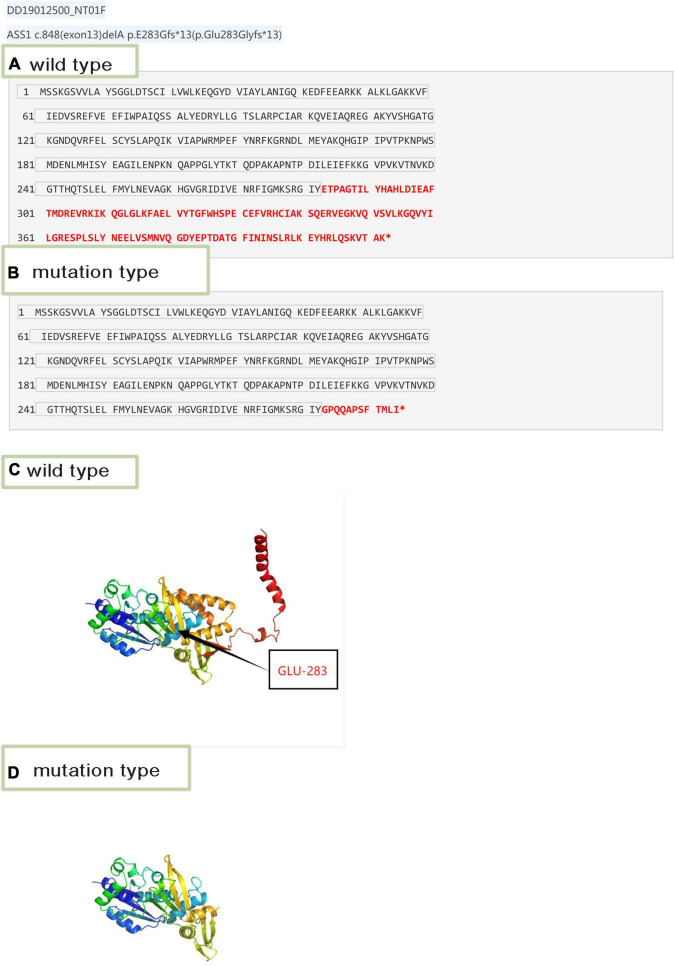
Amino acid sequence and 3D structure. **(A)** Wild-type amino acid sequence of *ASS1*:c.848delA. **(B)** Mutation type amino acid sequence of *ASS1*:c.848delA. **(C)** 3D structure of wild-type of *ASS1* (p.Glu283 Glyfs*13). **(D)** 3D structure of mutation type of *ASS1(*p.Glu283 Glyfs*13).

The 3D structure of the novel mutant c.848delA p.Glu283 Glyfs*13 was predicted (Figure 3). According to the amino acid sequence (Figures 3A,B), the novel mutation c.848delA, p.Glu283 Glyfs*13 changed glutamic acid to glycine; this frameshift mutation resulted in formation of a truncated protein (Figures 3C,D). The truncated proteins did not contain an off-PP-loop motif, which is necessary for maintaining ASS1 protein function. Therefore, the novel mutations c.848delA and p.Glu283 Glyfs*13 caused CTLN1.

**FIGURE 4:**
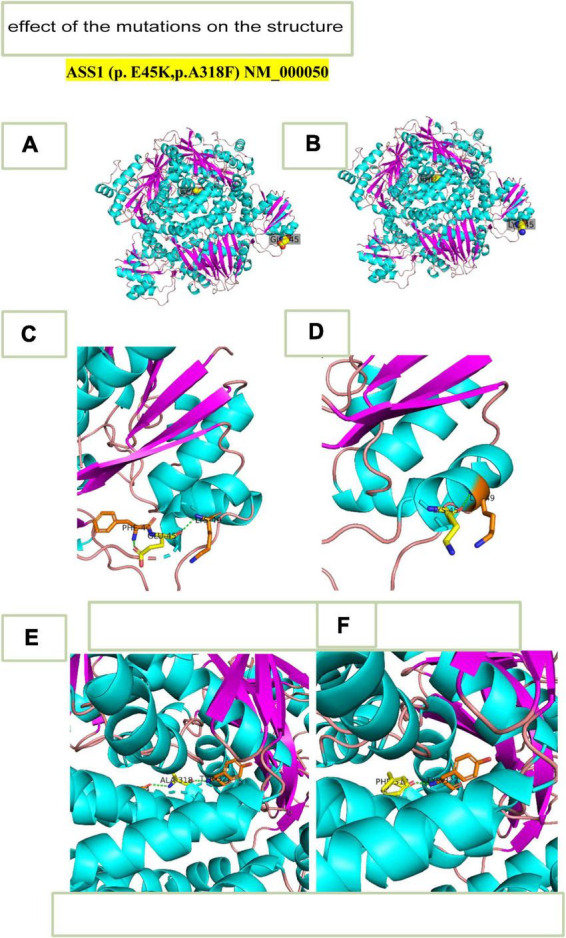
3D- structure. **(A)** 3D-structure of wild-type *ASS1* (p.E45K,p.A318F). **(B)** 3D structure of mutation *ASS1* (p.E45K,p.A318F). **(C)** 3D-structure of wild-type *ASS1* (p.E45K). **(D)** 3D structure of mutation type ASS1 (p.E45K). **(E)** 3D structure of wild-type *ASS1*(p.A318F). **(F)** 3D structure mutation type *ASS1* (p.A318F).

We predicted the 3D structure of the wild mutation (Figure 4A) and the novel mutants c.133G > A,p.Glu45Lys and c.952_953 delinsTT, p.Ala318Phe (Figure 4B). The c.133G > A,p.Glu45Lys mutation changed glutamic acid to lysine (Figures 4C,D), and c.952_953 delinsTT, p.Ala318Phe changed alanine to phenylalanine (Figures 4E,F). Based on these results, we evaluated the relationship between the translational products and the disease and alterations in the protein structural properties. The double hydrogen bond before the mutation was changed to a single hydrogen bond, which decreased the stability of the ASS1 protein and affected protein synthesis.

### Relation between ammonia and citrulline levels and clinical phenotypes

**FIGURE 5:**
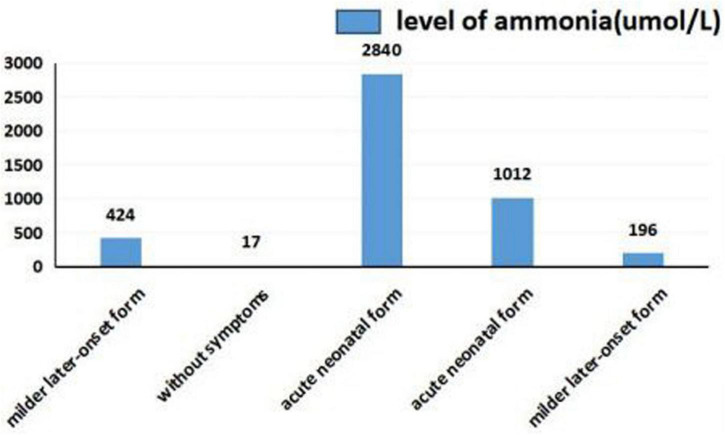
Ammonia levels in different clinical phenotypes.

**FIGURE 6:**
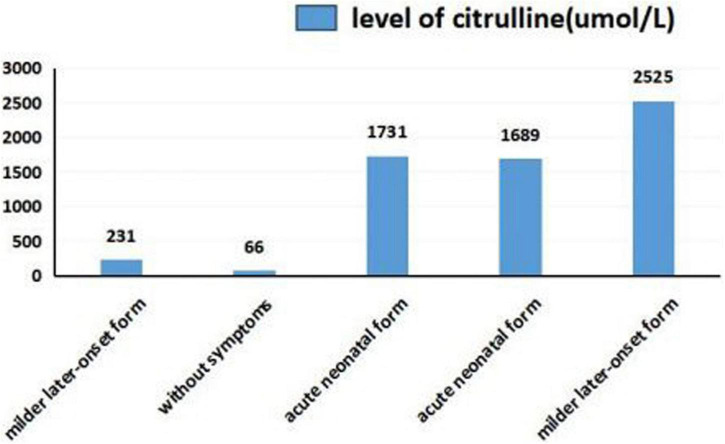
Citrulline levels in different clinical phenotypes.

As shown in Figures 5, 6, we summarized the characteristics of the five patients as follow: the blood ammonia levels were very high in patients with the neonatal disease form, whereas those in other forms, particularly asymptomatic and delayed onset CTLN1, were relatively low (Figure 5), as opposed to citrulline levels (Figure 6).

### Characteristics of citrullinemia type I in Chinese children

According to a literature review, 20 cases of CTLN1 have been reported. The characteristics of these cases are summarized in Tables 5, 6 (8–22).

**TABLE 5:** Clinical presentations and biochemical investigations of patients described in the literature.

Patient no.	Sex	Age of onset	Clinical presentation	Conscious disturbance or hyperspasmia	Vomiting or feeding difficulties	Highest level of ammonia	Highest level of citrulline	Outcome	References
1	F	1 y 3 m	Late-onset form	Yes	Yes	160	928.77	Moderate	(8)
2	F	2 d	Neonatal form	Yes	Yes	670	1,577	Died	(9)
3	–	2 d	Neonatal form	Yes	Yes	286	487	Died	(10)
4	M	1 Y	Late-onset form	–	–	91	961	Moderate	(11)
5	F	4 d	Neonatal form	Yes	Yes	231	1,085	Died	(11)
6	F	1 y 5 m	Late-onset form	–	–	126	653	Moderate	(12)
7	M	2 d	Neonatal form	Yes	Yes	–	2,513	Died	(13)
8	M	–	Mild form	No	No	111	17	Well	(14)
9	F	3 m	Late-onset form	No	Yes	–	311	Moderate	(15)
10	M	3 y	Mild form	No	No	90	70	Well	(16)
11	M	2 d	Late-onset form	Yes	Yes	1,692	2,563	Moderate	(17)
12	F	1 m	Late-onset form	–	–	23	1,924	Died	(18)
13	F	6 d	Neonatal form	Yes	Yes	–	1,621	Died	(19)
14	F	2 d	Neonatal form	Yes	Yes	398	3,188	Moderate	(20)
15	F	4 d	Neonatal form	Yes	Yes	77	173	Moderate	(20)
16	M	1 d	Neonatal form	Yes	Yes	366	2,977	Moderate	(20)
17	F	2 d	Neonatal form	Yes	Yes	2,077	2,092	Moderate	(20)
18	F	7 d	Neonatal form	Yes	Yes	280	3,188	Moderate	(20)
19	F	8 d	Neonatal form	Yes	Yes	371	2,329	Moderate	(21)
20	M	2 Y	Late-onset form	No	Yes	118	1,593	Moderate	(22)

**TABLE 6:** Genetic investigations of patients described in the literature.

Patient no.	Clinical presentation	Mutation 1			Mutation 2			References
		Location	cDNA	Protein	Location	cDNA	Protein	
1	Late-onset form	Exon 13	c.847G > A	Glu283lys	Exon 14	c.1009T > C	Cys337Arg	(8)
2	Neonatal form	Exon 13	c.951delT	F317LfsX375	Exon 14	c.1087C > T	Arg363Trp	(9)
3	Neonatal form	Exon 6	c.380G > A	Arg127Gln	Exon 6	c.380G > A	Arg127Gln	(10)
4	Mild form	Intron 4	c.174 + 1G > A	–	Exon 7	c.422 T > C	Val141Gly	(11)
5	Neonatal form	Intron 11	c.773 + 1G > A	–	Exon 12	c.793C > T	Arg265Cys	(11)
6	Late-onset	Exon 5	c.236C > T	Ser79Phe	Exon 7	c.431C > G	Pro144Arg	(12)
7	Neonatal form	Exon 13	c.970G > A	Gly324Ser	Exon 13	c.970G > A	Gly324Ser	(13)
8	Mild form	Exon 3	c.53C > T	Ser18Leu	Exon 15	c.1168G > A	Gly390Arg	(14)
9	Late-onset form	Exon 7	c.431C > G	Pro144Arg	Exon 14	c.1087C > T	Arg363Trp	(15)
10	Mild form	Intron 11	c.773 + 4A > C	–	Intron 11	c.773 + 4A > C	–	(16)
11	Late-onset form	–	–	–	–	–	–	(17)
12	Late-onset form	Exon 7	c.968C > T	Thr323Ile	Exon 7	c.937C > A	Leu313Met	(18)
13	Neonatal form	Intron 15–16	c. 1194 - 2 A¿G	–	Exon 3	–	–	(19)
14	Neonatal form	Exon 5	C.257G¿A	Arg86His	Intron 6–7	c.421-2A > G	–	(20)
15	Neonatal form	Exon 5	c.332C > T	Ala111Val	Exon 5	c.288C > G	No change	(20)
16	Neonatal form	Exon 5	c.291C > G	Cys97Trp	Exon 15	c.1168G > A	Gly390Arg	(20)
17	Neonatal form	Intron 6–7	c.421-2A > G	–	Exon 14	c.981-1018del38	H327Qfs*33	(20)
18	Neonatal form	Exon 5	c.256C > T	Arg86Cys	Exon 9	c.577G > A	Gly193Arg	(20)
19	Neonatal form	Exon 7	c.469C > T	Arg157Cys	Exon 8	c.552C > A	Asn184Lys	(21)
20	Late-onset form	Exon 7	c.470 G > A	Arg157His	Exon 9	c. 577 G > A	Gly193Arg	(22)

## Discussion

CTLN1 is a rare congenital metabolic disease caused by mutations in *ASS1*. Because of the different activities of argininosuccinic acid synthetase, the clinical presentation of CTLN1 is heterogeneous.

We evaluated five patients diagnosed with CTLN1; the clinical phenotype included three neonates, one late-onset, and one asymptomatic type. Most cases reported in the literature were the neonatal form, followed by the late-onset form. Our results are consistent with those of previous studies (23, 24).

We found that ammonia levels were higher in the neonatal form than in other forms, whereas this pattern was not observed for citrulline. However, we could not statistically analyze these results because the sample size was too small. To date, there have been no studies of the relationship between early blood ammonia and citrulline levels and the phenotype of CTLN1. We found that higher blood ammonia levels led to more severe clinical symptoms. The main reason for this result is that the pathogenesis of CTLN1 involves a high blood ammonia level, followed by an energy crisis in the brain tissue and subsequent cerebral edema. Thus, the blood ammonia level determines the severity of cerebral edema, which may be an independent risk factor for predicting the prognosis of CTLN1. Genetic testing and tandem mass spectrometry have only commonly performed in the region in recent years. Because of economic constraints and the lack of gene-tandem mass spectrometry 5 or 6 years ago, many children died from “unexplained encephalopathy” in the neonatal period, leaving CTLN1 undiagnosed. This is one reason why the sample size was small in this study. Confirmation of blood ammonia as an independent predictor of the phenotype of CTLN1 requires a large sample size and further development of gene and tandem mass spectrometry.

The novel mutations affected ASS1 function by changing the protein tertiary conformation in different manners. The genotype and phenotype correlations of CTLN1 remain unclear (6). The effects of the Gly324Ser and c.1128–6_1188dup67 mutations were mild. Nevertheless, the Gly324Ser mutation was associated with the acute neonatal and neonatal forms. In Iran (7), many patients with classic CTLN1 possessed mutations in *ASS1* (c.1168G > A; p.Gly390Arg), which led to poor prognosis including coma or death. In China, c.1168G > A mutations have been detected in patients with mild disease. The clinical presentation was also not identical in patients with the same genotype, possibly because the variations in most of the Chinese population are compound heterozygous mutations, making it difficult to analyze the genotype–phenotype relationship. To determine this relationship, larger sample sizes should be evaluated and further genetic testing should be performed. Further studies of the genotype, phenotype, and enzymes may reveal this correlation.

In China, CTLN1 is considered as a rare disease, whereas in other countries such as Iran (7) where consanguineous marriages occur, the incidence of CTLN1 has greatly increased. Therefore, advocating for healthy birth and postnatal care is important for preventing hereditary diseases.

Because of economic constraints and the lack of gene-tandem mass spectrometry 5 or 6 years ago, many children died from unexplained encephalopathy in the neonatal period, leaving CTLN1 undiagnosed and providing a small sample size for this study. Thus, multicenter studies of larger sample sizes are needed to define the relationship between the genotype and phenotype with increased accuracy and reliability.

## Data availability statement

The datasets presented in this study can be found in online repositories. The names of the repository/repositories and accession number(s) can be found in the article/supplementary material.

## Ethics statement

The studies involving human participants were reviewed and approved by the Ethics Committee of the First Affiliated Hospital of USTC with registration number: 2022-RH-232. Written informed consent to participate in this study was provided by the participants’ legal guardian. Written informed consent was obtained from the individual(s), and minor(s)’ legal guardian/next of kin, for the publication of any potentially identifiable images or data included in this article.

## Author contributions

MC and MX conceptualized and designed the study and contributed substantially to the wrote of this manuscript. MX conducted the study, coordinated and supervised data collection, and contributed to the drafting of this manuscript. MC performed the data management, conducted the statistical analyses, and drafted the results in the manuscript. Both authors approved the final manuscript as submitted and agreed to be accountable for all aspects of the work.

## Footnotes

1
http://code.google.com/p/cutadapt/

2
https://www.sentieon.com/

3
http://annovar.openbioinformatics.org/en/latest/

4
https://swissmodel.expasy.org/interactive
